# Evaluation of a social marketing intervention promoting oral rehydration salts in Burundi

**DOI:** 10.1186/1471-2458-11-155

**Published:** 2011-03-08

**Authors:** Sethson Kassegne, Megan B Kays, Jerome Nzohabonayo

**Affiliations:** 1 Population Services International/Benin, B.P. 08-0876 Tri Postal Cotonou, Benin; 2 Population Services International, 1120 Nineteenth Street NW, Suite 600, Washington, D.C. 20036, USA; 3 Population Services International/Burundi, B.P. 1474 Bujumbura, Republique du Burundi

## Abstract

**Background:**

Diarrhea is the second leading cause of death for children under five in Burundi; however, use of oral rehydration salts (ORS), the recommended first-line treatment, remains low. In 2004, PSI/Burundi launched a social marketing intervention to promote ORASEL among caregivers of children under five; the product was relaunched in 2006 with a new flavor. This study evaluates the intervention after the ORASEL relaunch, which included mass media and interpersonal communication activities. The study looks at trends in ORASEL use in Burundi and in behavioral determinants that may be related to its use.

**Methods:**

In 2006 and 2007, PSI conducted household surveys among Burundian females of reproductive age (15-49). Both surveys used a two-stage sampling process to select 30 households in each of 115 rural and urban collines throughout the nation. Survey respondents were asked about diarrhea treatment-related behavior; key behavioral determinants; and exposure to the ORASEL intervention. Data were analyzed to identify trends over time, characteristics of ORASEL users, and associations between exposure to the intervention and changes in ORASEL use and related behavioral determinants.

**Results:**

ORASEL use among caregivers at their children's last diarrheal episode increased significantly from 20% in 2006 to 30% in 2007, and there were also desirable changes in several behavioral determinants associated with ORASEL use. Evaluation analysis showed that a higher level of exposure to the social marketing campaign was associated with greater use of ORASEL and with significant improvements in perceived availability, knowledge of the signs of diarrhea and dehydration, social support, and self-efficacy.

**Conclusions:**

ORS use can be improved through social marketing and educational campaigns that make the public aware of the availability of the product, encourage dialogue about its use, and increase skills and confidence relating to correct product preparation and administration. Further interventions in Burundi and elsewhere should promote ORS through a variety of mass media and interpersonal communication channels, and should be rigorously evaluated in the context of the total market for diarrhea treatment products.

## Background

Diarrheal diseases are one of the leading causes of mortality among children under the age of five, resulting in 16.7% of infant and child deaths globally [1]. In Burundi, diarrhea is the third leading cause of death for children under five after malaria and pneumonia, according to national health center data [2].

According to a Multiple Indicator Cluster Survey (MICS) conducted in 2005, 20.6% of children under five had an episode of diarrhea within the past two weeks [3].

These numbers may actually underestimate the cases of diarrhea, as noted in the last Demographic and Health Surveys from Burundi [4]. Underestimates of diarrhea may result from people misunderstanding the definition of diarrhea or not perceiving diarrhea as an illness. Studies in various African countries have found that caregivers believe diarrhea to be caused by teething, exposure to cold, poor-quality milk or breastmilk, consumption of candy or uncooked food, or supernatural forces [3,5-7]. Many feel that diarrhea is a natural phenomenon and not a serious issue unless they notice additional symptoms, such as fever or listlessness [5,7-9].

The majority of diarrheal deaths are due to dehydration as a result of the loss of fluids. Dehydration can be treated most effectively with a sugar-salt solution known as oral rehydration therapy (ORT), which includes both homemade solutions and commercially marketed products - oral rehydration salts (ORS). The World Health Organization (WHO) recommends providing low osmolarity ORS to children under five as the primary strategy for reducing deaths from diarrhea and as the first line of care, in addition to continued provision of food and fluids [10]. Despite the efficacy of ORS, an analysis of DHS data from 40 countries demonstrated that overall uptake of ORS has increased by less than 1% per year from 1986 to 2003 and has declined in many countries in recent years [11].

There are limited studies on diarrhea treatment and ORS use in Burundi. The 2005 MICS found that 36.5% received ORT of any type, and 30.4% received ORS [3]. The DHS survey in 1987 found that 30% received ORT of any type, 8% received a homemade oral rehydration solution, and 38% received antibiotics or medicinal plants [4]. Thus, there have been modest increases over time, but ORS adoption remains low.

More recent studies from other African countries have found high levels of antibiotic use to treat diarrhea and correspondingly lower levels of ORS use and fluid intake. Researchers in Nigeria reported that 68% of a cohort of 80 women administered antibiotics to children who had diarrhea, and just 23% used a sugar-salt solution [5]. Another Nigerian study found that traditional medicine was the first-line treatment for diarrhea, and that less than one in ten female caregivers provided ORS [12]. In Kenya, a longitudinal study found that 45% of caregivers used antibiotics and just 13% used ORS [13]. Many caregivers reported relying on medicinal plants and herbs or supernatural treatments [8,13]. In addition to inappropriate treatment, many caregivers reduced fluids and foods provided to the child [13,14].

Low use of ORS may be linked to caregivers' perceptions of the cause of the diarrhea and thus the appropriate route of treatment; for example, mothers believing that diarrhea was related to teething were less likely to seek treatment [6,7,14]. Treatment providers may also be complicit in providing inappropriate care; in Tanzania, drug store employees recommended antibiotics 44% of the time and ORS and fluid intake just 29% of the time [15] and in Egypt, private providers were less likely to prescribe ORS [16].

A 1992 study found that availability of ORS in Burundi was not an issue, as 90% of the population had access to it. However, fewer than one-third used it. The study concluded that educational programs were needed to increase ORS acceptance and use [17]. The lack of published research about the effectiveness of such interventions in Burundi leaves programmers and funders with an insufficient evidence base for determining how to encourage greater ORS uptake.

The purpose of this study is to evaluate a social marketing intervention that promoted ORASEL, a low osmolarity ORS product developed by FDC Limited and branded and distributed by Population Services International (PSI). Its goal was to examine the effect of the program on ORASEL use; impact in terms of reduction of mortality due to diarrheal disease was not measured. The study evaluates the intensified intervention following the relaunch of the ORASEL product in 2006.

Specifically, the study looks at trends in ORASEL use in Burundi and in behavioral determinants (environmental and psychosocial factors that may influence behavior) that may be related to its use; identifies which behavioral determinants are significantly associated with ORASEL use; and examines correlations between exposure to the social marketing intervention and changes in behavior and behavioral determinants over time.

## Methods

### Theoretical framework

**Figure 1:**
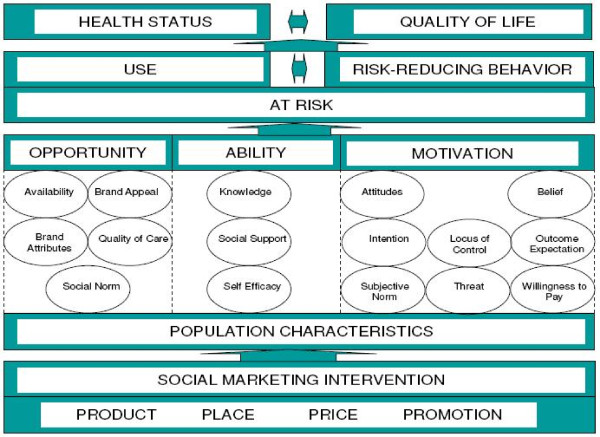
**The Performance Framework for Social Marketing (PERForM), with the PSI Behavior Change Framework as a Component of the Second Level**. [SUBMITTED IN SEPARATE FILE PER AUTHOR INSTRUCTIONS].

This study was guided by PSI's internal frameworks for behavior change and health impact, the Performance Framework for Social Marketing (PERForM) and the PSI Behavior Change Framework (Figure 1). PERForM describes a set of theoretical pathways through which social marketing interventions can potentially influence behaviors that have health-related consequences [18]. The goal of the social marketing intervention is to improve the health status or quality of life of at-risk individuals by influencing behavioral determinants in ways that lead to greater use of protective products or services and/or to increased risk-reducing behavior.

The PERForM framework distinguishes between two different types of determinants that influence behaviors of interest: population characteristics and mutable behavioral determinants. By addressing the behavioral determinants thought to have the greatest effect on the behaviors of interest, a social marketing intervention can indirectly bring about changes in those behaviors. Within the second level of the PERForM framework, the PSI Behavior Change Framework categorizes behavioral determinants into 16 summary constructs. As Figure 1 indicates, these constructs can be thought of as either opportunity, ability, or motivation determinants, a framework initially introduced by Rothschild (1999) [19]. Opportunity determinants encompass institutional or structural factors affecting whether or not someone performs a desired behavior; ability determinants relate to the skills or proficiencies needed to perform the behavior; and motivation determinants influence the person's desire to perform the behavior. The sixteen determinants are drawn from health behavior theories including the Health Belief Model [20] and the Theory of Reasoned Action [21], as well as marketing theory [22].

This study focuses on ten determinants thought to be relevant to caregivers' use of ORS, selected based on their statistical relevance (demonstrated in other studies on diarrhea treatment) and programmatic relevance: availability of ORASEL, brand appeal of ORASEL, knowledge of diarrhea and diarrhea treatment, social support for diarrhea treatment, self-efficacy to correctly prepare and administer ORASEL, intention to use ORASEL to prevent dehydration in a child with diarrhea, locus of control for diarrhea treatment, outcome expectation of the efficacy of ORASEL for preventing dehydration, threat related to the severity of diarrhea, and willingness to pay for ORASEL.

### Programmatic intervention

PSI/Burundi implemented a social marketing intervention to promote ORASEL with funding from USAID. The primary objective of the program was to increase the use of ORASEL for treatment of diarrhea in children under five.

In 2005, PSI/Burundi moved to update its ORS product to meet UNICEF's new reduced osmolarity guidelines and to change its flavor, adding an orange taste (as field research indicated that children did not like the taste of the original formula ORASEL). There was limited stock of ORASEL in May to September of 2006, and the new orange-flavored ORASEL was launched in October 2006. The price of ORASEL was the same throughout the intervention period, at 50 Burundi Francs (about $0.05).

The program utilized mass media and interpersonal communication activities in rural and urban areas from 2004 to 2007 to promote ORASEL. Four radio spots were developed and broadcast: two on the importance and use of ORS, specifically ORASEL, and two addressing the causes and consequences of diarrhea, including its severity for children under five and how to prevent it. After the launch of the orange-flavored ORASEL, an additional radio spot was developed to advertise the new product. These spots aired on six radio stations a total of 4,994 times.

To complement the radio campaign, community outreach activities were conducted at schools and health centers. Additionally, 1,914 health workers, vendors, and pharmacy employees were trained in the promotion and use of ORASEL. Educational and promotional materials, including posters, brochures, t-shirts, stickers, backpacks, pens, notebooks, and bibs, were distributed through the outreach activities. These activities were intensified on a national scale after the launch of the new orange-flavored ORASEL. In total, community outreach exposed a total of 572,674 people to the intervention.

### Ethical Review

Population Services International has conducted formative and evaluative research on health behaviors among diverse populations worldwide for over thirty years, and complies with local standards for protection of human subjects in countries in which it operates.

This study was conducted by researchers who were trained in courses certified by the Office of Human Resources Protections on the Code of Standards and Ethics for Survey Research. All participants in the study underwent an informed consent process detailing the purpose of the study, that their participation was voluntary, and that their answers were confidential; only those who verbally consented to participation were included in the study. Researchers protected the anonymity and confidentiality of the respondents by not collecting identifying information and by ensuring that only members of the research team had access to the raw data, which was kept in locked file cabinets.

Burundi does not have a formally registered ethical review board that addresses child survival research. Local governmental authorities were informed of and approved the research studies. PSI established its own Review Ethics Board in 2009, after these data were collected.

### Sample and design

In 2006 and 2007, PSI conducted household surveys among Burundian females of reproductive age (15-49). The 2006 survey included modules on HIV as well as diarrhea treatment, and the 2007 survey included modules on malaria, water treatment, and diarrhea treatment. In both, the module on diarrhea treatment was presented last.

For the purposes of the study presented in this paper, we analyzed data from a study subgroup comprised of women who were caregivers of at least one child under the age of five (from overall samples of women of reproductive age). In 2006, it was assumed that all children under five had experienced a diarrheal episode at least once, thus all caregivers of children under five were included in the subsample. In 2007, caregivers were asked if their child had experienced an episode of diarrhea since birth, and only those who said yes were included in the subsample. Non-caregivers and those whose children had not had diarrhea were excluded. A caregiver was defined as a person who provides primary care to a child.

Sample sizes were calculated based on estimates needed to measure changes in health behaviors over time with a confidence level of 95% for diarrhea treatment with ORS and condom use in 2006 and for insecticide treated net use, water treatment with a chlorine-based product, and diarrhea treatment with ORS in 2007, with an effect size of 1.5 and an assumption of a 10% non-response rate and 1.6 females of reproductive age per household. The calculations indicated a need for 4,440 survey respondents in 2006 from 2,775 households and 5,535 survey respondents in 2007 from 3,459 households. A larger sample size was needed in 2007 as additional data were sought on urban households in malaria-endemic zones. The calculations were not adjusted for clustering effects.

Both surveys used the same sampling methodology, including a two-stage sampling process. Burundi's 2,639 collines comprised the sampling frame. (The nation's 17 provinces are subdivided into communes, which are further subdivided into collines.) In the first stage, 115 collines were randomly selected from both rural and urban areas in each province with a probability proportional to the sizes of the provinces and collines. In the second stage, 30 households were randomly selected from a full listing of households each colline. (In the 2007 survey, additional households were sampled from urban collines to achieve the needed sample size.) Each household was contacted and a record was made of the members of the household. All female household members aged 15-49 were then asked if they would serve as survey participants and those who consented were interviewed.

Of the 3,728 women surveyed in 2006, 2,499 met the criteria for our study (were a caregiver of a child under five). In 2007, 2,101 of 5,408 survey respondents met the criteria (were a caregiver of a child under five and reported that at least one of their children had ever had diarrhea).

### Measures

The full questionnaire was designed by PSI/Burundi and was pretested before each round. The questionnaire measured information about household and sociodemographic characteristics. The diarrhea treatment-specific portion of the questionnaire measured key diarrhea treatment behavioral indicators; opportunity, ability, and motivation (OAM) determinants of behavior; and exposure to the ORASEL intervention.

The primary outcome of interest, use of ORASEL during a child's last episode of diarrhea, was measured as a binary yes/no variable. The OAM determinants were measured using a variety of single statements that were also treated as binary yes/no variables. These are reported as percentages of respondents who agreed with the statements.

Program exposure was measured by two yes/no questions, one asking if the respondent had ever attended an activity relating to the preparation and use of ORASEL and the other asking if the respondent had seen ORASEL promotional information or heard ORASEL advertisements during the previous three months. Respondents who reported being exposed to neither or one of the communication channels (media or community outreach) were classified as having no/low exposure to the program. Those who reported being exposed to both communication channels were classified as having high exposure to the program.

### Data collection

Interviewers were recruited and trained in interviewing skills. The trainings lasted five days and included a pretest of interviewing skills before interviewers were authorized to collect data. Trainings were conducted by the Institute of Statistics and Economic Studies of Burundi (ISTEEBU) in 2006 and by PSI/Burundi in 2007. Data collection took place in June 2006 and July 2007 (the rainy season where diarrhea would be most likely is October to December). During data collection, PSI/Burundi provided supervision and oversight to improve data quality. In each selected household, interviewers approached all eligible women and explained the purpose of the study. A verbal consent script was used to make all respondents aware of the goals of the study and of their rights as participants. Women who verbally agreed were interviewed.

### Analysis

Four separate analyses were conducted. First, frequencies were run to describe the population characteristics of the two samples and they were compared for any significant differences using bivariate statistics (chi-square). Second, UNIANOVA was used to produce adjusted proportions and to monitor trends over time in ORASEL use, behavioral determinants, and exposure to the intervention (Table 1). Third, logistic regression was employed to conduct a segmentation analysis comparing ORASEL users and non-users in the 2007 study population. All sociodemographic and behavioral variables were entered into the model, and non-significant items were dropped then re-entered one by one until the Wald statistic was <1.0. The remaining non-significant items were dropped. The significant items are presented in Table 2. UNIANOVA was used to provide the adjusted proportions, with other significant variables acting as controls. Finally, an evaluation analysis was run using UNIANOVA to compare the first round group and two second round groups (no/low exposure to the intervention and high exposure to the intervention). ORASEL use and the behavioral determinants that showed significant changes over time in the monitoring analysis were included and tested for associations with no/low exposure and high exposure (Table 3). All analyses were conducted using SPSS 10. The analyses controlled for sociodemographic determinants as appropriate to mitigate differences in the 2006 and 2007 study populations.

**Table 1:** Trends over time in ORASEL use, behavioral determinants, and exposure to the ORASEL social marketing intervention among female caregivers of children under five in Burundi

INDICATORS	June 2006 N = 2,499	July 2007 N = 2,101	P-value
BEHAVIOR/USE	%	%	
ORASEL use during child's last episode of diarrhea	20.0	30.0	0.000

			

**OPPORTUNITY**	%	%	

***Availability***			

Thinks that ORASEL is available to everyone at an affordable price	44.4	40.9	0.114

Thinks that the scarcity of ORASEL is a barrier to its use	74.1	63.5	0.000

Thinks that the price of ORASEL is an obstacle to its use	36.0	13.4	0.000

***Brand Appeal***			

Thinks that many people believe ORASEL to be the best of the ORS products usually distributed in health centers	93.6	88.8	0.000

Thinks that people prefer ORASEL to other ORS at health centers	88.7	87.1	0.303

Thinks that the brand of ORS used to treat dehydration is not important	27.9	18.2	0.000

			

**ABILITY**	%	%	

***Knowledge***			

Knows at least two signs of diarrhea	47.2	56.1	0.000

Knows that dehydration is the primary cause of death in children with diarrhea	92.0	95.1	0.001

Knows at least one sign of severe dehydration from diarrhea	85.0	89.1	0.001

Knows at least one means of treating dehydration from diarrhea	87.2	81.2	0.000

***Social Support***			

Has discussed the use of ORASEL to prevent dehydration from diarrhea	28.2	38.5	0.000

***Self-Efficacy***			

Feels capable of preparing and administering ORASEL solution to a child with diarrhea	26.8	38.5	0.000

			

**MOTIVATION**	%	%	

***Intention***			

Would buy ORASEL to treat her children in the future	93.5	95.4	0.291

***Locus of Control***			

Thinks that diarrhea is inevitable among children under five	72.9	81.2	0.000

***Outcome Expectation***			

Thinks that ORASEL is very effective against dehydration from diarrhea	95.3	97.5	0.004

Thinks that ORASEL does not have any effect on dehydration among children with diarrhea	23.3	19.3	0.022

***Risk Perception (Perceived Severity of Threat)***			

Thinks that treatment of diarrhea can be completely managed at home without needing to go to a health center	22.3	15.0	0.000

Thinks that diarrhea must be treated in a health center	93.1	93.8	0.473

Thinks that after giving ORASEL to a child for dehydration, it is necessary to bring him/her to a health center to treat the cause of the diarrhea	90.5	94.7	0.000

***Willingness to Pay***	*Burundi* *Francs*	*Burundi* *Francs*	

	204.2	229.5	0.000

			

**EXPOSURE**	%	%	

Has attended a demonstration on ORASEL use	21.4	26.8	0.001

Has heard or seen ORASEL commercials in the last three months	84.6	88.0	0.010

The UNIANOVA model controlled for age, residence (rural/urban), marital status, religion, occupation, and level of education. Willingness to pay was adjusted to reflect for inflation.

**Table 2:** Indicators significantly associated with use of ORASEL for a child's last diarrheal episode among female caregivers of children under five in Burundi, 2007

INDICATORS	ORASEL Users N = 610	ORASEL Non-Users N = 1,491	OR	P-value
**OPPORTUNITY**	%	%		

***Availability***				

Thinks that ORASEL is available to everyone at an affordable price	56.2	45.8	1.8	0.002

Thinks that the scarcity of ORASEL is a barrier to its use	52.2	64.2	0.5	0.000

***Brand Appeal***				

Thinks that people prefer ORASEL to other ORS at health centers	89.7	83.2	2.0	0.005

				

**ABILITY**	%	%		

***Social Support***				

Has discussed the use of ORASEL to prevent dehydration from diarrhea	55.9	46.0	1.7	0.002

***Self-Efficacy***				

Feels capable of preparing and administering ORASEL solution to a child with diarrhea	73.2	26.8	14.0	0.000

The logistic regression controlled for age, residence (rural/urban), marital status, religion, occupation, and level of education.

Hosmer & Lemeshow Test: χ^2 ^= 13,4865; ddl = 7; p < 0,0611.

Tests of the specification of the model: χ^2 ^= 644,377; ddl = 5; p < 0,001; Cox & Snell R^2 ^= 0,400; Nagelkerke R^2 ^= 0,541.

**Table 3:** Associations between exposure to the ORASEL social marketing intervention and changes in ORASEL use and related behavioral determinants among female caregivers of children under five in Burundi

INDICATORS	First round (2006) N = 2,499 (54.3%)	Second round (2007)		
		
		No/Low exposure N = 1,584 (34.4%)	High exposure N = 517 (11.2%)	P-value
**BEHAVIOR/USE**				

ORASEL use during child's last episode of diarrhea	20.5^a^	12.8^b^	75.3^c^	0.000

				

**OPPORTUNITY**				

***Availability***				

Thinks that the scarcity of ORASEL is a barrier to its use	73.8^a^	73.5^a^	40.8^b^	0.000

Thinks that the price of ORASEL is an obstacle to its use	35.9^a^	15.1^b^	10.1^c^	0.000

***Brand Appeal***				

Thinks that many people believe ORASEL to be the best of the ORS products usually distributed in health centers	93.7^a^	85.6^b^	95.5^a^	0.000

Thinks that the brand of ORS used to treat dehydration is not important	27.9^a^	17.9^b^	19.2^b^	0.000

				

**ABILITY**				

***Knowledge***				

Knows at least two signs of diarrhea	47.3^a^	52.1^b^	66.9^c^	0.000

Knows that dehydration is the primary cause of death in children with diarrhea	92.1^a^	94.6^b^	96.3^b^	0.003

Knows at least one sign of severe dehydration from diarrhea	85.1^a^	86.8^a^	95.3^b^	0.000

Knows at least one means of treating dehydration from diarrhea	87.3^a^	78.3^b^	88.7^a^	0.000

***Social Support***				

Has discussed the use of ORASEL to prevent dehydration from diarrhea	28.8^a^	20.4^b^	86.2^c^	0.000

***Self-Efficacy***				

Feels capable of preparing and administering ORASEL solution to a child with diarrhea	27.5^a^	13.3^b^	88.1^c^	0.000

				

**MOTIVATION**				

***Locus of Control***				

Thinks that diarrhea is inevitable among children under five	72.9^a^	81.5^b^	80.2^b^	0.000

***Outcome Expectation***				

Thinks that ORASEL is very effective against dehydration from diarrhea	95.3^a^	97.4^b^	97.7^b^	0.017

Thinks that ORASEL does not have any effect on dehydration among children with diarrhea	23.3^a^	17.7^b^	22.5^a^	0.010

***Risk Perception (Perceived Severity of Threat)***				

Thinks that treatment of diarrhea can be completely managed at home without needing to go to a health center	22.3^a^	15.3^b^	14.4^b^	0.000

Thinks that after giving ORASEL to a child for dehydration, it is necessary to bring him/her to a health center to treat the cause of the diarrhea	90.5^a^	94.7^b^	94.9^b^	0.000

***Willingness to Pay***	*Burundi* *Francs*	*Burundi* *Francs*	*Burundi* *Francs*	

	204.3^a^	226.0^b^	238.7^b^	0.000

The UNIANOVA controlled for for age, residence (rural/urban), marital status, religion, occupation, and level of education. Willingness to pay was adjusted to reflect for inflation.

The letters a, b, and c indicate statistically significant differences between successive categories (the reference/first round category and the two second round exposure categories). When two categories share the same letter, they are not significantly different from one another.

## Results

### Sample description

There were no significant differences between 2006 and 2007 survey respondents in terms of marital status, occupation, education, or age. (Data not shown.) The majority of women in both rounds were married (85.7% in 2006 and 84.6% in 2007), stated their occupation as farming/agriculture (73.6% in 2006 and 71.4% in 2007), and had a primary school education or less (85.7% in 2006 and 85.3% in 2007). The average age for both cohorts was 29 years.

2006 and 2007 respondents differed in regard to religion and place of residence (urban/rural). (Data not shown.) In 2006, 93% of women reported themselves to be Christian, whereas in 2007, 91% did so (p < .05). The 2006 sample did not have as many urban residents as the 2007 sample (26.9% vs. 67.5%, p < .001). This difference reflects the additional sampling of urban households in 2007 for the malaria component of the survey. Religion and place of residence were controlled for in the analyses.

### Trends over time

Table 1 presents trends over time in ORASEL use, behavioral determinants thought to be related to diarrhea treatment behavior, and exposure to the ORASEL intervention.

There was a statistically significant increase from 2006 to 2007 in the key behavior of interest, use of ORASEL during a child's last diarrheal episode, from 20% in 2006 to 30% in 2007 (p < .001).

Indicators for two opportunity-related determinants, availability and brand appeal, showed significant changes over time. For availability, the percentage of caregivers who thought that ORASEL's scarcity and price were barriers to use decreased significantly (p < .001 for both). Brand appeal indicators showed mixed changes. The percentage of caregivers who thought that many people believe ORASEL to be the best ORS product distributed at health centers decreased from 93.6% to 88.8% (p < .001), but there was no significant difference in perceptions that people prefer ORASEL to other ORS products at health centers. There was an almost 10% decrease in the proportion of respondents who felt that the brand of ORS used to treat dehydration was not important (p < .001).

The majority of the ability-related determinants showed significant improvements between 2006 and 2007 in terms of knowledge, social support, and self-efficacy. All of the knowledge indicators except one increased significantly over time. The increases were for knowledge of at least two signs of diarrhea (p < .001), knowledge that dehydration is the leading cause of death for children with diarrhea (p < .001), and knowledge of at least one sign of severe dehydration (p < .01). In contrast, the percentage of caregivers who knew at least one treatment method for dehydration decreased significantly, from 87.2% to 81.2% (p < .001).

Social support improved over time, with an increase in the percentage of caregivers who had discussed ORASEL use for the prevention of dehydration with peers (28.2% vs. 38.5%, p < .001). Likewise, self-efficacy improved over time, with an increase in the percentage of caregivers who reported that they felt capable of preparing ORASEL and administering it to their children (26.8% vs. 33.9%, p < .001).

There were mixed trends among the motivation-related determinants, which included intention, locus of control, outcome expectation, risk perception, and willingness to pay. Intention, operationalized as being ready to buy ORASEL to treat children's diarrhea in the future, was quite high at the first round (93.5%) and did not change significantly over time. Caregiver locus of control decreased over time, with more caregivers in 2007 expressing the belief that diarrhea is inevitable among children under the age of five (72.9% vs. 81.2%, p < .001). On the other hand, both indicators measuring outcome expectation showed improvements over time. At the second round, more caregivers felt that ORASEL was very effective against dehydration (p < .01) and fewer felt that ORASEL did not have any effect on dehydration (p < .05).

One measure of perceived severity of diarrhea, the percentage of caregivers who felt that treatment of diarrhea could be completely managed at home without seeking care at a health center, dropped from 22.3% to 15.0% (p < .001). Another, the percentage who felt that after administration of ORASEL, children should be taken to a health center to receive diarrhea treatment, increased from 90.5% to 94.7% (p < .001).

Willingness to pay, as measured by the maximum stated amount that a caregiver would be willing to spend on one packet of ORASEL, increased significantly from 204 to 230 Burundi Francs (p < .001). (This indicator was only measured for ORASEL, not for other diarrhea treatment products.)

Exposure to ORASEL messaging increased significantly from 2006 to 2007. The percentage of caregivers who had attended a demonstration on ORASEL use increased from 21.4% to 26.8% (p < .01), and who had heard an ORASEL radio spot in the last three months increased from 84.6% to 88.0% (p < .05).

### Determinants associated with ORASEL use

Table 2 shows which behavioral determinants were found to be significantly associated with ORASEL use among 2007 survey respondents. The target audience (female caregivers of children under five) was segmented based on the behavior of interest, use of ORASEL at a child's last diarrheal episode. Those who performed the behavior (used ORASEL) were compared to those who did not. Only indicators that showed significant differences between the users and nonusers are included in the table.

The segmentation analysis found differences in indicators measuring two opportunity determinants, availability and brand appeal, and two ability determinants, social support and self-efficacy. No motivation-related indicators were significantly associated with ORASEL use.

Regarding availability, those who felt that ORASEL was sold at an affordable price were 1.8 times as likely to be ORASEL users (p < .01), and those who felt that the scarcity of ORASEL was a barrier to use were half as likely to be ORASEL users (p < .001). Regarding brand appeal, ORASEL users were twice as likely as non-users to think that people prefer ORASEL to other ORS products at health centers (p < .01).

In terms of social support, those who reported having discussed ORASEL use with others were 1.7 times as likely to have used ORASEL at a child's last diarrheal episode (p < .01). Finally, ORASEL users were far more likely to feel capable of preparing and administering ORASEL than non-users (odds ratio, 14.0; p < .001).

### Effects of program exposure

Table 3 shows associations between exposure to the ORASEL social marketing intervention and changes in ORASEL use and related behavioral determinants, and Table 4 shows the pairwise comparisons. Only indicators that changed significantly over time in the monitoring table were included in Table 3. Three exposure groups were compared: first round (2006); no/low exposure (exposed to no ORASEL communications or exposed to just one type of media, either media or community outreach) at the second round; and high exposure (exposed to both media and community outreach) at the second round.

**Table 4:** Pairwise comparisons for associations between exposure to the ORASEL social marketing intervention and changes in ORASEL use and related behavioral factors among female caregivers of children under five in Burundi presented in table 3

INDICATORS	First Round and No/Low exposure	First round and High exposure	No/Low exposure and High exposure
**BEHAVIOR/USE**			

ORASEL use during child's last episode of diarrhea	0.000	0.000	0.000

			

**OPPORTUNITY**			

***Availability***			

Thinks that the scarcity of ORASEL is a barrier to its use	0.888	0.000	0.000

Thinks that the price of ORASEL is an obstacle to its use	0.000	0.000	0.043

***Brand Appeal***			

Thinks that many people believe ORASEL to be the best of the ORS products usually distributed in health centers	0.000	0.302	0.000

Thinks that the brand of ORS used to treat dehydration is not important	0.000	0.000	0.580

			

**ABILITY**			

***Knowledge***			

Knows at least two signs of diarrhea	0.020	0.000	0.000

Knows that dehydration is the primary cause of death in children with diarrhea	0.012	0.002	0.244

Knows at least one sign of severe dehydration from diarrhea	0.228	0.000	0.000

Knows at least one means of treating dehydration from diarrhea	0.000	0.483	0.000

***Social Support***			

Has discussed the use of ORASEL to prevent dehydration from diarrhea	0.000	0.000	0.000

***Self-Efficacy***			

Feels capable of preparing and administering ORASEL solution to a child with diarrhea	0.000	0.000	0.000

			

**MOTIVATION**			

***Locus of Control***			

Thinks that diarrhea is inevitable among children under five	0.000	0.002	0.580

***Outcome Expectation***			

Thinks that ORASEL is very effective against dehydration from diarrhea	0.013	0.022	0.773

Thinks that ORASEL does not have any effect on dehydration among children with diarrhea	0.004	0.734	0.047

***Risk Perception (Perceived Severity of Threat)***			

Thinks that treatment of diarrhea can be completely managed at home without needing to go to a health center	0.000	0.000	0.707

Thinks that after giving ORASEL to a child for dehydration, it is necessary to bring him/her to a health center to treat the cause of the diarrhea	0.000	0.003	0.883

***Willingness to Pay***	0.000	0.000	0.088

ORASEL use differed significantly across the three exposure categories. At the first round, 20.5% of caregivers used ORASEL at last diarrheal episode. At the second round, 12.8% of caregivers with no/low exposure to the intervention did so, while 75.3% of caregivers with high exposure did so (p < .001 overall).

Perceived availability, an opportunity variable, improved significantly with exposure to the program. Roughly three-quarters of those in the first round and no/low exposure groups felt that the scarcity of ORASEL was an obstacle to its use, as compared to only 40% of those highly exposed (p < .001 overall). While both second round groups were less likely than the first round group to believe that the price of ORASEL was a barrier to its use, the high exposure group had a greater decline for this indicator (first round, 35.9%; no/low exposure, 15.1%; high exposure, 10.1%; p < .001 overall).

Brand appeal did not show an improvement based on exposure. For the item "thinks that many people believe ORASEL to be the best of the ORS products usually distributed in health centers," there was no significant difference between the first round and high exposure, though respondents with no/low exposure at the second round were significantly less likely to agree (p < .001 overall). This suggests that the belief diminished over time but that the effect was mitigated with high exposure to the intervention. Although the proportion of women who thought that the brand of ORS used was not important decreased from the first round to the second round, there was no significant difference between the no/low and high exposure groups at the second round.

Two of the knowledge items changed significantly with high exposure to ORASEL programming. The proportion of respondents who knew at least two signs of diarrhea increased significantly across exposure groups (47.3% to 52.1% to 66.9% for first round, no/low, and high, respectively; p < .001 overall). Knowledge that dehydration is the primary cause of death among children with diarrhea improved between the first and second round, but there was no significant difference between the no/low and high exposure groups at the second round. Knowledge of at least one sign of severe dehydration in children with diarrhea did not significantly differ between the first round and no/low exposure, but significantly improved among those with high exposure at the second round (p < .001 overall). Knowledge of at least one means of treating dehydration from diarrhea decreased significantly from the first to second round among those with no/low exposure to the ORASEL program, but not among those who were highly exposed.

Both social support and self-efficacy demonstrated associations with exposure, with significant decreases from the first round for the no/low exposure second round group and significant increases for the high exposure second round group. The indicator measuring social support for discussing ORASEL use with others increased from 28.8% at the first round to 86.2% at the second round for highly exposed respondents, and the indicator measuring self-efficacy for preparing and administering ORASEL increased from 27.5% at the first round to 88.1% at the second round for the same group (p < .001 for both, overall).

While all of the motivation determinants improved between the first and second round, only one item showed a significant difference between the no/low exposure group and the high exposure group. The outcome expectation item "thinks that ORASEL does not have any effect on dehydration among children with diarrhea" decreased significantly from the first round to the second round for the no/low exposure group, but this decline appears to have been mitigated by high program exposure; the high exposure group did not significantly differ from the first round. Locus of control, outcome expectation regarding ORASEL's effectiveness in preventing dehydration, risk perception, and willingness to pay did not show any relationship to exposure at the second round, suggesting that although these improved over time, the changes were not associated with level of exposure to the ORASEL campaign.

## Discussion

The purpose of this study was to evaluate the effectiveness of PSI/Burundi's social marketing campaign to increase the use of ORASEL oral rehydration salts to treat children under the age of five. ORASEL use among caregivers at their children's last diarrheal episode increased from 2006 to 2007, and there were also desirable changes in several behavioral determinants thought to be related to the use of ORASEL. A segmentation analysis of the 2007 caregiver sample found significant associations between ORASEL use and the following behavioral determinants: perceived availability (including perceived affordability), brand appeal, social support for discussing ORASEL with others, and self-efficacy for preparing and administering ORASEL. The evaluation analysis showed that greater exposure to the social marketing campaign was associated with increased use of ORASEL and with improvements in perceived availability, knowledge of the signs of diarrhea and dehydration, social support, and self-efficacy.

This study had several limitations. First, because of budget constraints, the surveys from which the data were drawn were not designed to focus specifically on diarrhea treatment and ORS, but encompassed a broad range of health problems including HIV and malaria. The sampling strategies were based on epidemiological patterns for those health problems, leading to samples that included disproportionate representation of young people and urban residents. To address this issue, the analyses presented here controlled for age and place of residence, as well as other sociodemographic characteristics. However, there may have been other differences in the study populations that were not measured that may have influenced the results. Also, the length of the questionnaire increased for the second round, which may have increased respondent fatigue, thus reducing data quality, though none of the data collectors noted that this was an issue. Additionally, there may have been changes in other determinants of ORASEL use that we were unable to detect with our sample size.

A second limitation was the time frame specified for the behavior of interest (ORASEL use). In research that addresses how caregivers treat their children's diarrhea, the standard recall period is two weeks. Most studies limit the sample to caregivers who had a child with diarrhea in the last two weeks, and ask about the caregiver's treatment practices for that child. In our study, the 2006 survey did not include questions about whether the caregivers' children had experienced diarrhea. Instead, based on the prevalence of diarrhea among children under the age of five in Burundi, it was assumed that every caregiver of one or more children under five must have at least one child under five who had experienced diarrhea at some point since birth. The 2007 survey used having had a diarrheal episode since birth as a screening question. The reference period for treatment was thus less specific than the usual reference period of two weeks. Instead, caregivers were asked if they used ORASEL the last time one of their children had diarrhea.

Increasing the recall period in this way could have resulted in inaccurate reporting from respondents, namely in underestimating both diarrhea incidence and ORASEL use; the first round PSI survey found that just 20% used ORS, compared with the 2005 MICS, which found that 30.4% used ORS. This underestimation is likely due to failure to recall the diarrhea treatment used. However, as this failure to recall should be present in both rounds of the survey and would not be expected to vary from round to round, the increase observed in ORS use is highly unlikely to be the result of poor recall or misreporting.

A third limitation is the absence of a control group, without which it is difficult to quantify the effect of other environmental forces on improving ORS use. However, as ORT use only increased 6% over a period of 18 years (from the 1987 DHS to the 2005 MICS), it is unlikely that ORS use would increase by 10% within the space of one year without external intervention. As we know of no other major programmatic interventions promoting ORS in these areas or any major social or environmental factors occurring during this time period, the dramatic increase in ORS use can most likely be attributed to PSI's intervention.

In conjunction with the lack of a control group, the follow-up group only included two exposure measures: no/low exposure and high exposure. Unfortunately, given the intensity of the campaign there were not enough caregivers reporting no exposure to be able to statistically compare them with those reporting just one type of exposure and those reporting both interpersonal communication (IPC) and media, which makes it difficult to assess to what degree ORS uptake may have naturally increased without the program.

Another limitation of the study is that it addressed only ORS use for diarrhea treatment, and did not measure use of alternative treatments or the order of treatments. This limits our ability to track changes in these over time or to see if ORS was adopted as the first treatment or was used after other treatments or in conjunction with inappropriate treatments.

A final limitation was that the first round survey was conducted after the intervention began, so the reference group was already well exposed to some of the messaging. However, the product was relaunched shortly after the first round survey, with an intensified media campaign to promote its use, so the data reflect that programmatic phase.

Despite its limitations, our study demonstrates a clear link between exposure to a social marketing campaign to promote an ORS product and increased use of that product. The findings suggest that using mass media and IPC can significantly improve determinants of ORS use, namely perceived availability, social support for discussing ORS, and self-efficacy for preparing and administering ORS, and thus can subsequently increase ORS use itself. The change to the new orange-flavored ORASEL may have also increased use, as it improved caregiver and child acceptance of the product.

It is important to note that the intervention was most effective when mass media and IPC were combined; an intervention that offers one or the other of these may not show the same level of effectiveness in changing behavior. This can present a programmatic challenge, as reaching a critical mass with IPC may be very resource-intensive.

Our results are consistent with those obtained by other researchers. Kenya et al. demonstrated a positive effect of communication campaigns on knowledge and acceptability of ORS within a district in Kenya [23]. A study of an intervention providing direct distribution of ORS in Guinea-Bissau showed that availability of the product and educational sessions improved ORS uptake [24]. Ross-Degnan et al. concluded that similar educational campaigns can influence pharmacists, as an intervention for Kenyan pharmacists was linked to increased sale of ORS and a decline in the use of antibiotics to treat diarrhea [25].

Further studies of ORS use in Burundi and of other ORS social marketing interventions should examine the entire market for ORS products and homemade oral rehydration therapy (ORT). This study did not include information on use of non-ORASEL ORS, and while the promotion of ORASEL was brand-specific, it may nonetheless have had a halo effect of improving ORS use generally. This issue is of particular interest since caregivers did not necessarily think that it mattered which brand of ORS was used. Another important question is how a campaign promoting ORS or ORT affects uptake of the various competing products within the diarrhea treatment sector. It is especially important to monitor the use of antibiotic treatment, to see if the promotion of ORS results in a decline in use of antibiotics or other inappropriate first-line treatments.

More studies looking at the effects of ORS/diarrhea education on locus of control and risk perception are also warranted. In this study, more caregivers in the second round survey felt that diarrhea was inevitable and that it required professional treatment rather than home management. These findings may reflect an increased awareness of the potential for diarrhea to be life-threatening for children under five, but may be counterproductive to ORS interventions that promote home management of diarrhea as well as to safe water and hygiene interventions that seek to prevent diarrhea. Although neither of these determinants was significantly different between users and non-users of ORS, more research is needed to better understand caregivers' perceptions of ORS/diarrhea education, the effects on prevention and treatment of diarrheal disease, and how to best message to increase confidence in home-based care.

## Conclusions

The study results presented here, coupled with other researchers' findings, suggest that ORS use can be improved through social marketing campaigns that make the public aware of the availability of the product, encourage dialogue about its use, and increase skills and confidence relating to correct product preparation and administration. Further interventions in Burundi and elsewhere should promote ORS through a variety of mass media and IPC channels, and should be rigorously evaluated in the context of the total market for diarrhea treatment products.

## Competing interests

The authors declare that they have no competing interests.

## Authors' contributions

At the time of the study, SK was the Regional Researcher for West and Central Africa, based in Cotonou, Benin. MBK was an Associate Researcher for PSI, based in Washington, D.C. JN was the Research Manager for PSI/Burundi. JN and SK developed the study design, implemented the study, and analyzed the results. SK reviewed and finalized the report. SK wrote the first draft of the paper excluding the discussion, and MK provided translation, editing, and content assistance, and wrote the conclusion. All authors read and approved the final manuscript.

## Pre-publication history

The pre-publication history for this paper can be accessed here:

http://www.biomedcentral.com/1471-2458/11/155/prepub
